# Antimicrobial stewardship: attempting to preserve a strategic resource

**DOI:** 10.3402/jchimp.v1i2.7209

**Published:** 2011-07-18

**Authors:** Trevor Van Schooneveld

**Affiliations:** Department of Internal Medicine, Division of Infectious Disease, University of Nebraska Medical Center, Omaha, NE, USA

**Keywords:** antimicrobial stewardship, resistance, pre-authorization, audit and feedback

## Abstract

Antimicrobials hold a unique place in our drug armamentarium. Unfortunately the increase in resistance among both gram-positive and gram-negative pathogens coupled with a lack of new antimicrobial agents is threatening our ability to treat infections. Antimicrobial use is the driving force behind this rise in resistance and much of this use is suboptimal. Antimicrobial stewardship programs (ASP) have been advocated as a strategy to improve antimicrobial use. The goals of ASP are to improve patient outcomes while minimizing toxicity and selection for resistant strains by assisting in the selection of the correct agent, right dose, and best duration. Two major strategies for ASP exist: restriction/pre-authorization that controls use at the time of ordering and audit and feedback that reviews ordered antimicrobials and makes suggestions for improvement. Both strategies have some limitations, but have been effective at achieving stewardship goals. Other supplemental strategies such as education, clinical prediction rules, biomarkers, clinical decision support software, and institutional guidelines have been effective at improving antimicrobial use. The most effective antimicrobial stewardship programs have employed multiple strategies to impact antimicrobial use. Using these strategies stewardship programs have been able to decrease antimicrobial use, the spread of resistant pathogens, the incidence of *C. difficile* infection, pharmacy costs, and improved patient outcomes.

Antimicrobial agents have radically altered medical care and life expectancy over the last 70 years. The introduction of antimicrobials was associated with a rapid decline in infectious disease mortality and an improvement in life expectancy (1). The introduction of agents that effectively treated infections not only improved outcomes of infectious diseases, but also paved the way for treatment of oncology and transplant patients. Antimicrobials hold a unique place in our drug armamentarium as the only drugs that lose effectiveness with increased use. They are also considered ‘societal’ drugs as antimicrobial use and misuse can benefit or harm patients who do not receive them (2).

## The rising tide of resistance

Unfortunately, resistance to antimicrobials is increasing throughout the world and now threatens our ability to treat even simple infections. The ICU rates of methicillin-resistant *Staphylococcus aureus* (MRSA) are greater than 60%, and MRSA has become the primary pathogen causing skin and soft tissue infections in US emergency departments (3, 4). Resistance in gram-negative pathogens has increased as well, with a multi-center survey from 1999 to 2008 showing fluoroquinolone resistance in *E. coli* increasing from 4.1 to 31.8% in 2008. A similar pattern has been noted with other agents and Enterobacteriaceae species (5). A major concern has been the emergence of carbapenemases, which are enzymes that hydrolyze all beta-lactam antibiotics including carbapenems. The most commonly described carbepenemase in the United States is the *Klebsiella pneumoniae* carbapenemase (KPC), which has caused numerous outbreaks, is detectable in most states, and has become endemic in certain cities and countries (6–10). Another recently described carbapenemase, the New Delhi metallo-beta-lactamase-1 (NDM-1), was initially detected in India, Pakistan, and the United Kingdom. However, it has since been detected on most continents, suggesting rapid spread of this resistance mechanism (11–13).

## No new drugs

Antimicrobial resistance not only limits therapeutic options but also results in increased patient morbidity, mortality, and health care costs (14–18). The lack of new antimicrobials to treat these pathogens is creating a crisis that may return medicine to the pre-antibiotic era. Antimicrobial development has been stagnant for years and even if suggested policy changes are implemented the effect will not be seen for years (19, 20). As new agents will not be readily available, clinicians must learn to make the best use of antimicrobials.

## Antimicrobial use and the selection for resistance

Antimicrobial use is the key driver of antimicrobial resistance. In European Union countries, the increasing use of penicillin type antibiotics is associated with increasing resistance in *S. pneumoniae* (21, 22). Similar findings of increased antibiotic use associated with resistance have been described on the national level, community level, hospital level, and individual patient level (23–27). While antimicrobial use is unavoidable, there is strong evidence that the use is suboptimal. It is estimated that up to 50% of all antimicrobial use in the hospital is inappropriate (28). One institution evaluated antimicrobial use over a 2-week period and classified 30% of all antibiotic days as unnecessary and 58% of all patients received at least 1 day of unneeded treatment (29). Similar findings have been described in the outpatient setting with an estimated 6.5 million antibiotic prescriptions yearly for common colds in children (30, 31).

## Antimicrobial stewardship

**Fig. 1:**
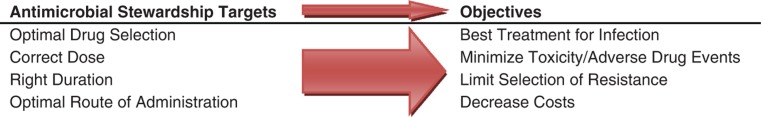
Antimicrobial stewardship targets and objectives.

A proposed solution to the combined problems of increasing antibiotic resistance, the dwindling number of antimicrobial agents, and the suboptimal use of antibiotics in clinical practice is the strategy of antimicrobial stewardship. The term ‘stewardship’ describes the careful or responsible management of a valued entity entrusted to one's care. Antimicrobial agents should be viewed as a shared resource that must be managed with an eye to preservation of their use for future generations (32). Antimicrobial stewardship is defined as interventions to improve the appropriate use of antimicrobials through promotion of optimal agent selection, dosing, duration, and route of administration. The objectives of antimicrobial stewardship are focused on achieving optimal clinical outcomes while minimizing toxicity, adverse events, and selection for antimicrobial resistant strains. Cost reduction may be a result of stewardship programs but should not be an overarching goal (Fig. 1).

Comprehensive guidelines for development of an institutional antimicrobial stewardship program have been published by the Society for Healthcare Epidemiology of America (SHEA) and Infectious Diseases Society of America (IDSA) (28). These guidelines describe two core strategies and several supplemental strategies for improving antimicrobial use. These core strategies are not mutually exclusive and may be integrated to varying degrees in different settings.

## Restriction and pre-authorization

The first strategy is a ‘front end’ strategy that targets antibiotic use at the time it is ordered and consists of either formulary restriction and/or pre-authorization. The strategy of restricting the antibiotic availability has been highly effective at reducing use and costs, but is unpopular among clinicians, and the impact on resistance has been variable (33–35). A survey of 22 institutions found that those that implemented a program of carbapenem restriction experienced a statistically significant reduction in both carbepenem use and *Psuedomonas aeruginosa* resistance to carbapenems compared to those institutions that allowed unrestricted use of carbapenems (36).

Restrictions have also been effective in prevention of *Clostridium difficile* infection (CDI). The major risk event for CDI is the disruption of normal enteric flora by antimicrobial agents (37). At one center, the use of a bundled set of interventions including pre-authorization resulted in reduced targeted antibiotic use of 41% and nosocomial CDI of 71% over 5 years (38). Similarly, an outbreak of severe CDI was controlled only after restrictions of high-risk antimicrobials (39).

The major drawback to strategies centered on antibiotic restriction and pre-authorization is that antibiotic use is driven to other agents that select for resistance. For example, Rahal and colleagues decreased cephalosporins use 80% through using restrictions and noted a 44% decrease in ESBL-producing *Klebsiella*. However, imipenem use increased 141% and imipenem-resistant Pseudomonas increased 69% (40). The phenomenon of restriction driving use to another agent has been described as ‘squeezing the balloon’ and may mitigate some or all of the benefits of a restrictive strategy (41, 42). Another disadvantage of these strategies is that they may not be well accepted and ordering physicians may attempt to circumvent them (43).

## Prospective audit and feedback

The other major stewardship strategy advocated is prospective audit and feedback. This strategy maintains clinician authority for initial antimicrobial choice, but reviews the antimicrobial selection in real-time providing ‘unsolicited’ advice. Advantages of this strategy include the ability to provide education at the point of intervention and customization of the intervention to any patient group, drug, or syndrome. For example, one institution provided all patients on broad spectrum therapy with customized recommendations for streamlining or discontinuation. This intervention did not change the hospital antibiogram but resulted in a 28% decrease in the use of broad-spectrum agents at that institution (44). At another center, a multidisciplinary team made customized antibiotic recommendations and calculated a cost savings of $2602 per intervention (45).

A disadvantage of audit and feedback is increased time and clinical expertise compared to other interventions. Another disadvantage is that the antibiotic recommendation is usually considered optional, though rates of compliance with recommendations have been reported to be between 70 and 90% (45–48).

## Other interventions

A variety of other elements may be integrated into an antimicrobial stewardship program including use of guidelines/clinical pathways, education, use of biomarkers, computerized decision support, antimicrobial cycling, use of combination therapy, streamlining/de-escalation protocols, antibiotic dose optimization, and parenteral to oral conversion protocols (Table 1) (28). An extensive review of each of these topics is beyond the scope of this manuscript, but some unique aspects of these elements will be highlighted. The use of guidelines and clinical pathways has been effective in changing clinician behavior. A multi-center study randomized hospitals to the use of a clinical pathway for community-acquired pneumonia or usual care. The investigators demonstrated a decrease in rates of hospitalization, length of stay, and duration of intravenous therapy, with an increased use of narrow spectrum antibiotics and no change in the rates of complications, readmission, or mortality (49). In the surgical setting, one tertiary care center mandated use of a surgical antimicrobial prophylaxis order set and noted significant improvements in surgical quality of care measures including appropriateness of choice, duration of use, and dose selection; all at a cost savings to the institution (50).

**Table 1:** Antimicrobial stewardship strategies

Strategy	Intervention	Comments
Formulary restriction	Only certain agents available for use	Effective, minimal effort to maintain, usurps prescriber autonomy, ‘squeezing the balloon’
Pre-authorization	Certain agents available if specific criteria are met	Effective, allows education, requires designated call person, creates tension between prescriber and stewardship program, clinician may circumvent the system
Audit and feedback	Review of ordered antimicrobials or culture results	Customizable, educational, maintains prescriber autonomy, recommendations specific to clinical situation, optional, time intensive, requires reviewer with broad knowledge
Guidelines/clinical pathways	Development of institution specific recommendations for management of specific disease states	Ability to standardize practice and meet quality measures; customized to institutional needs, resources and formulary, compliance usually not mandatory; perception of ‘cookbook medicine’
Education	Education to clinicians regarding appropriate antimicrobial use	Alters behavioral patterns, improves compliance with guidelines, decreased misuse, poor efficacy of passive education, diminishing effect over time, rotation of personal means repeated education needed
Biomarkers	Use of laboratory information (procalcitonin, etc.) to assist in therapy decisions	Objective marker, well validated in certain situations, must be used with overall patient evaluation, false positive and negative results can occur, clinicians must be able to interpret to use correctly
Antibiotic cycling	Scheduled removal and substitution of antimicrobial or class	May decrease resistance rates, compliance difficult due to patient allergies, national guidelines, and adverse events
Combination therapy	Use of two or more agents to prevent resistance and improve clinical outcomes	Evidence for improvement in clinical outcomes is variable, minimal data to support decrease in resistance, increased risk of allergy and toxicity, increased cost
Streamlining/de-escalation	Narrowing of broad spectrum therapy when no pathogen isolated or targeting of pathogen isolated	Decreases antimicrobial use and costs, allows narrowest effective therapy if culture data available, possible under-treatment if no culture data acquired, treatment of colonizers
Dose optimization	Use of pharmacokinetic-pharmacodynamic (PK-PD) parameters to optimize dosing	Improves likelihood of achieving PK-PD targets, may allow use of agents against resistant pathogens, may decrease selection of resistant strains, some data improves clinical outcomes, requires expertise and time, may be limited by microbiologic data available (MIC), limited data on clinical outcomes
Computerized decision support	Use of information technology to assist clinicians in antimicrobial decision making	Can be integrated into EHR, integrates local data for customized recommendations, requires significant technology and personnel input to set up, alert fatigue, requires constant updating
IV to oral conversion	Agents with excellent oral bioavailability are switched from IV to oral	Decreases cost and need for IV access, may decrease length of stay, only certain agents have excellent oral bioavailability

Education is an important aspect of any stewardship program. While isolated educational interventions do not typically result in dramatic changes in practice, they can occasionally significantly alter use (51). In response to an outbreak of ESBL-producing *K. pneumoniae*, Tängdén et al. developed recommendations for empiric antimicrobial therapy and disseminated them at their institution (52). Following this intervention, cephalosporin use declined 48%, fluoroquinolone and carbapenem use were unchanged, piperacillin/tazobactam and penicillin G use increased, and the ESBL outbreak abated. Whether educational interventions can consistently achieve similar results remains unclear, but the coupling of education with other interventions is more likely to result in a significant impact. At a single hospital, the step-wise introduction of an antimicrobial order set, extensive education of clinicians, and feedback and modification of regimens resulted in significant changes in choice of antimicrobials, a cost savings of $913,236 over 2 years, and a decrease in resistant gram-negative pathogens and MRSA infections (53).

Clinical prediction rules and biomarkers, such as procalcitonin, have also been effective in optimizing antimicrobial use. Their use in varied settings has been associated with a significant decrease in antimicrobial use, antimicrobial duration, resistant pathogens, costs, and duration of hospital stay without any increase in mortality (54–60).

While single interventions have demonstrated efficacy, stewardship programs that use multiple integrated interventions are more likely to produce a significant impact. Carling and colleagues published their experience over 7 years using a combination of audit and feedback, education, selective reporting of sensitivities, formulary management, and automatic stop orders to decrease broad-spectrum antibiotic use 22%, despite a 15% increase in the acuity of patients (61). *Clostridium difficile* and infections due to resistant Enterobacteriaceae at their institution declined as well. The future of stewardship will likely include integration of multiple interventions using decision support software in the electronic health record. For example, one institution developed an ICU-based computerized antibiotic decision support system with web-based pre-approval and recommendations for antimicrobials based on patient specific culture data (62). While overall antimicrobial use was unchanged during the course of the study, *P. aeruginosa* susceptibility to imipenem, ceftazidime, and gentamicin significantly improved. As these technologies become more available and sophisticated, the integration of local data, customized institutional management algorithms, and electronic resources will further improve antimicrobial use.

To confront the increasingly resistant pathogens and lack of agents available to treat them, all stakeholders must be engaged in the process of antibiotic stewardship. Antimicrobial use in the community and long-term care facility must also be addressed, as suboptimal prescribing of antibiotics in these settings can result in the spread of resistant pathogens into hospitals (25, 63). Agriculture and veterinary use of antimicrobials also play a role in the propagation of resistant species and antimicrobial use in these sectors must be controlled. The science of antimicrobial stewardship must continue to be developed and refined; the most efficacious strategies need to be identified and widely adopted. All clinicians must come to understand the unique place antimicrobials hold in medicine and work to preserve their utility.
